# Vascular plant taxa occurrences in exotic woodland and in natural and production forests on the Islands of São Miguel, Terceira and Pico (Azores)

**DOI:** 10.3897/BDJ.11.e109082

**Published:** 2023-08-03

**Authors:** Lurdes Borges Silva, Patrícia Madeira, Diogo Pavão, Rui B Elias, Monica Moura, Luís Silva

**Affiliations:** 1 BIOPOLIS Program in Genomics, Biodiversity and Land Planning, CIBIO, Campus de Vairão, 4485-661, Vairão, Portugal BIOPOLIS Program in Genomics, Biodiversity and Land Planning, CIBIO, Campus de Vairão, 4485-661 Vairão Portugal; 2 UNESCO Chair – Land within Sea: Biodiversity & Sustainability in Atlantic Islands. University of the Azores, Rua da Mãe de Deus, 9500-321, Ponta Delgada, Portugal UNESCO Chair – Land within Sea: Biodiversity & Sustainability in Atlantic Islands. University of the Azores, Rua da Mãe de Deus, 9500-321 Ponta Delgada Portugal; 3 CIBIO, Research Center in Biodiversity and Genetic Resources, InBIO Associate Laboratory, Campus Ponta Delgada-Faculty of Sciences and Technology, University of the Azores, Rua da Mãe de Deus, 9500-321, Ponta Delgada, Portugal CIBIO, Research Center in Biodiversity and Genetic Resources, InBIO Associate Laboratory, Campus Ponta Delgada-Faculty of Sciences and Technology, University of the Azores, Rua da Mãe de Deus, 9500-321 Ponta Delgada Portugal; 4 Faculty of Sciences and Technology, University of the Azores, Rua da Mãe de Deus, 9500-321, Ponta Delgada, Portugal Faculty of Sciences and Technology, University of the Azores, Rua da Mãe de Deus, 9500-321 Ponta Delgada Portugal; 5 Faculty of Agricultural Sciences and Environmental, University of the Azores, Rua Capitão João d’Ávila – Pico da Urze, 9700‑042, Angra do Heroísmo, Portugal Faculty of Agricultural Sciences and Environmental, University of the Azores, Rua Capitão João d’Ávila – Pico da Urze, 9700‑042 Angra do Heroísmo Portugal; 6 cE3c - Center for Ecology, Evolution and Environmental Changes & CHANGE - Global Change and Sustainability Institute & Azorean Biodiversity Group, University of the Azores, 9700-042, Angra do Heroísmo, Portugal cE3c - Center for Ecology, Evolution and Environmental Changes & CHANGE - Global Change and Sustainability Institute & Azorean Biodiversity Group, University of the Azores, 9700-042 Angra do Heroísmo Portugal

**Keywords:** Azores, islands, Plantae, endemic, native, exotic woodland, natural forest, production forest, Magnoliophyta, Magnoliopsida, Liliopsida, Lycopodiophyta, Pinophyta, Pteridophyta, occurrences

## Abstract

**Background:**

The data presented here originated from field expeditions carried out between 2017 and 2018, within the framework of Forest-Eco^2^ project: "Towards an Ecological and Economic valorisation of the Azorean Forest". The project aimed to quantify the ecological value of the Azorean forests, including carbon accumulation and to design and propose measures that could further enhance forest sustainability. For that, 90 forest plots were sampled on three Azores islands - São Miguel, Terceira and Pico - equally distributed into natural forest, exotic woodland and production forest. The aim of this report is to further expand knowledge on biodiversity trends enclosed in the different forest types present in the Azores, by providing a list of the occurrences of the 105 different vascular plant taxa together with a brief characterisation of their origin and life-form.

**New information:**

We provide an inventory of indigenous and non-indigenous vascular plant taxa from 90 forest stands. A total of 105 taxa were identified and registered, belonging to 60 families, 91 genera, 101 species and four subspecies. A total of 35% of the taxa were endemic, 27% native and 38% non-indigenous, including 19% of invasive taxa. Endangered and vulnerable taxa were registered, including *Elaphoglossumhirtum* (Sw.) C.Chr., *Lactucawatsoniana* Trel. and others which were considered by the authors a priority for conservation (e.g. *Arceuthobiumazoricum* Wiens & Hawksw., *Bellisazorica* Hochst. ex Seub., *Saniculaazorica* Guthnick ex Seub., *Platantheramicrantha* (Hochst. ex Seub.) Schltr.). Our records provide detailed and updated knowledge of Azorean Forest flora and highlight the role of natural forests as indigenous plant diversity hotspots and exotic woodland as a source of invasive taxa within the Archipelago.

## Introduction

Studies on ecology and the distribution of organisms in a gradient of forest types, particularly with an emphasis on flora and species attributes, have been the focal point in many projects on forests ecosystems across the world (Rahbek 1995, Kessler 2000, Pavón et al. 2000, Grytnes 2003, Olthoff et al. 2016, Vinod et al. 2022) and also in the Azores Islands (Marcelino et al. 2013, Marcelino et al. 2014, Elias et al. 2016, Borges Silva L et al. 2022b).

Forests and woodlands constitute a striking and structuring element of the Azorean landscape. Occupying about 30% of the insular terrestrial territory (DRRF 2014), they harbour enormous terrestrial biodiversity, making them, in essence, critical habitats for plant species and for providing a wide range of ecosystem services (Matos et al. 2019, Borges Silva L et al. 2022b).

The flora of the Azores comprises about 4000 vascular plant taxa (Tracheobionta) (Silva et al. 2008, Silva et al. 2022, Borges Silva L et al. 2022a), including cultivated taxa; but excluding another 1397 taxa without a confirmed presence in the Azores. Distributed within several groups, namely Magnoliophyta, Pinophyta, Pteridophyta and Lycopodiophyta (Silva et al. 2008, Silva et al. 2010), approximately 133 species are considered as native and 101 endemic, distributed over 60 families, the vast majority of which only contain a single endemic taxon (Silva et al. 2008, Silva et al. 2022, Borges Silva L et al. 2022a). The families with the highest numbers of endemic taxa correspond to Asteraceae (13), Dryopteridaceae (8), Poaceae (12) and Apiaceae (7) (Silva et al. 2009, Silva et al. 2010, Moura et al. 2013, Borges Silva et al. 2016, Moura et al. 2018, Moura et al. 2019, Vieira et al. 2020, Elias et al. 2022). The remaining taxa consist of exotic plants, distributed over 200 families, 2901 considered as cultivated or at least imported, 322 as casual escapes and 578 as naturalized, of which 140 are invasive, occupying large extensions (Silva et al. 2008, Silva et al. 2022, Borges Silva L et al. 2022a).

As in many archipelagos, the Azorean primary forest was largely cleared and replaced by secondary forest and grassland (Matos et al. 2019, Pavão et al. 2021). Currently, land use in the Azores is dominated by pastures and agriculture (60%), planted or alien dominated forests (22%), with natural forests and vegetation representing 10% of the territory (DRRF 2014).

The Azorean production forest is dominated by a reduced number of species, including *Cryptomeriajaponica* D. Don (12,856 ha), *Eucalyptusglobulus* Labill. (3786 ha), *Pinuspinaster* Aiton (874 ha) and by non-productive exotic woodland occupying more than 30% of the forest areas, where *Pittosporumundulatum* Vent. is the dominant woody species, occupying 23,939 ha (Lourenço et al. 2011, DRRF 2014, Borges Silva et al. 2014, Borges Silva et al. 2017, Borges Silva et al. 2018).

Non-indigenous invasive species, such as *P.undulatum*, *Hedychiumgardnerianum* Sheppard ex Ker-Gawl., *Leycesteriaformosa* Wall., *Clethraarborea* Aiton, *Gunneratinctoria* (Molina) Mirb. and tree ferns, such as *Sphaeropteriscooperi* (F.Muell.) R.M.Tryon, *Sphaeropterismedullaris* Bernh and *Dicksoniaantarctica* Labill., currently threaten the conservation of endemic Azorean species and natural forests (Silva and Smith 2006, Silva et al. 2009, Costa et al. 2013, Borges Silva et al. 2014, Borges Silva et al. 2017, Silva and Beech 2017, Borges Silva et al. 2018).

The publication of updated species lists and suitable floristic data, makes available relevant information for the evaluation of the conservation status of species and ecosystems (Simões et al. 2021, Büttner et al. 2022).

This paper aims to publish a dataset of vascular plant occurrences in 90 Azorean forests, highlighting the importance of natural forests as indigenous plant diversity hotspots and of exotic woodland as a source of invasive taxa.

## General description

### Purpose

The purpose of this paper was to publish a dataset of vascular plant occurrences in three forest types (NF-Natural Forest, EW-Exotic Woodland and PF-Production Forest) on three islands of the Azores Archipelago (São Miguel, Terceira and Pico), already published in GBIF as a Darwin Core Archive.

## Project description

### Title

Vascular plant taxa occurrences in exotic woodland and in natural and production forests on the islands of São Miguel, Terceira, and Pico (Azores).

### Personnel

Lurdes Borges Silva, Patrícia Madeira, Diogo Pavão, Rui Bento Elias, Mónica Moura and Luís Silva.

### Study area description

**Figure 1.**
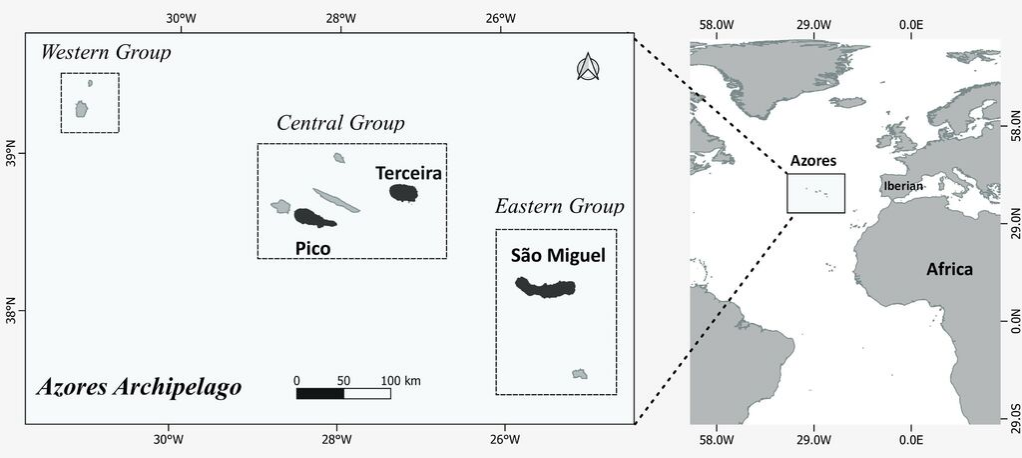
The Azores Archipelago with its location in the middle Atlantic (right panel) and Azorean Islands namely São Miguel, Terceira and Pico islands (left panel).

The Azores Archipelago is situated in the North Atlantic Ocean, between North America and Europe, about 1500 km west of mainland Portugal, roughly at 38°44'52''N, 31°32'16''W and 38°55'27''N, 25°0'36''W (Fig. 1). The Archipelago is formed by nine main islands and some small islets, all of them of volcanic origin. The islands are divided into three main groups: the western group (Corvo and Flores), the central group (Faial, Pico, Graciosa, São Jorge and Terceira) and the eastern group (São Miguel and Santa Maria). The climate in the Azores is temperate oceanic, with regular and abundant rainfall, high levels of relative humidity and persistent winds, mainly during winter and autumn (Azevedo et al. 2004).

The landscape of the islands is composed by a mosaic of habitats, ranging from herbaceous to arboreal and from natural to anthropogenic (Silva et al. 2008, Soares et al. 2021). The original landscape was strongly altered by replacing pristine and native forest areas with exotic tree plantations, crops, pastures and urban areas (Silva et al. 2008). During the last decades of the 20^th^ century, the reduction of native forest area was significant, with the clearing of large fragments, at mid- and high altitude, for pasture (Gaspar et al. 2008). *Pittosporumundulatum*, *Acaciamelanoxylon* R.Br. or *Eucalyptusglobulus* dominate most forest patches located in low- to mid-elevation areas. At higher altitudes, *Cryptomeriajaponica* dominates, along with the remaining stands of natural forests, particularly above 600 m a.s.l. (Elias et al. 2016, Borges Silva et al. 2017, Borges Silva et al. 2018, Dutra Silva et al. 2019). The natural vegetation includes diverse communities, namely coastal vegetation, coastal and inland wetlands, meadows, peat bogs and several types of native forests and scrubs. However, forests are the dominant natural vegetation type. In fact, before human settlement, laurel forests could have covered around 75% of Azorean islands (Elias et al. 2016). Currently, the native laurel forest comprises about 5% of the total surface of the Archipelago and has remained only at higher elevations and in inaccessible areas of the islands (Elias et al. 2016).

This research comprised three islands contributing with the largest forest areas: São Miguel Island with 745 km^2^, the highest elevation being 1105 m a.s.l. with an estimated age of 0.79 MY (millions of years) (Sibrant et al. 2015), Terceira Island with 400 km^2^, a maximum elevation of 1023 m a.s.l. and 0.39 MY (Hildenbrand et al. 2014) and Pico Island with an area of 447 km^2^, mostly occupied by a volcanoes reaching an altitude of 2351 m a.s.l. and an approximate age of 0.27 MY (Demand et al. 1982) (Fig. 1).

### Design description

**Figure 2.**
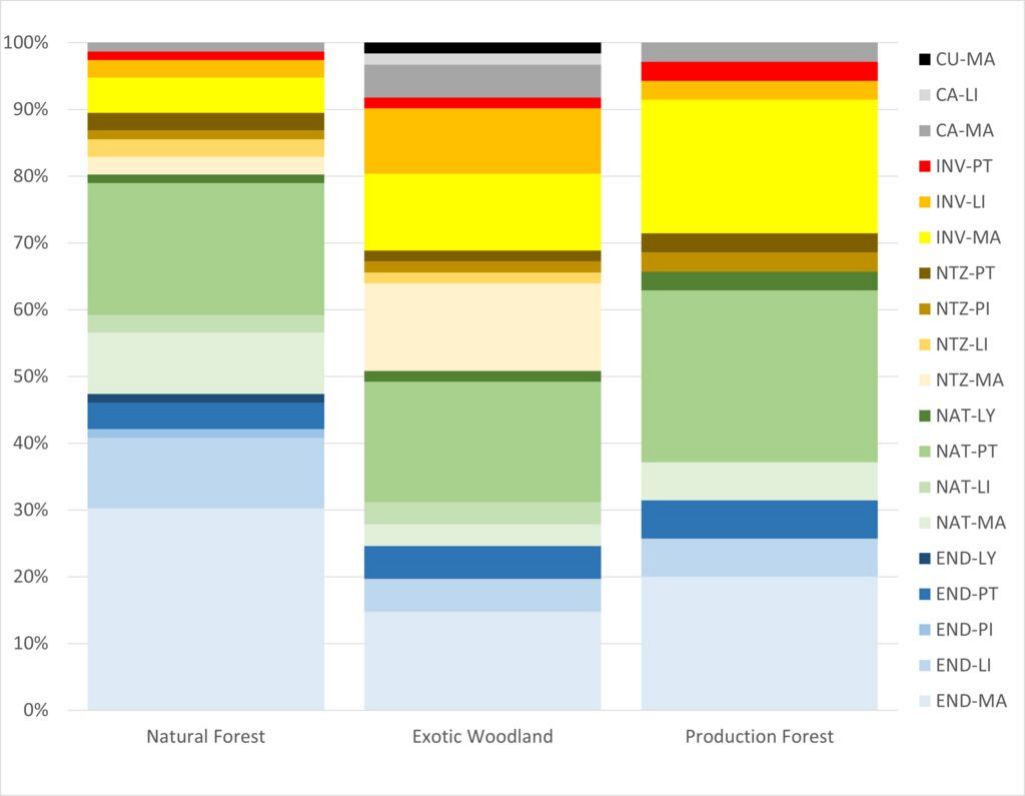
Categorisation of 105 Azorean vascular plant taxa, observed at 90 forests in the Azores, per forest type (Natural Forest, Exotic Woodland and Production Forest) and according to the five major groups of vascular plants (MA-Magnoliopsida, LI-Liliopsida, PI-Pinophyta, PT- Pteridophyta and LY-Lycopodiophyta,). Number of taxa in each category: END-Endemic (taxa only occurring in the Azores); NAT-Native (colonised the Azores without human intervention, also occurring in other regions); INV-Invasive; NTZ-Naturalised (with self-supporting populations); CA-casual (occasionally escaped from cultivation) and CU-Cultivated (Silva et al. 2010).

A total of 90 forests patches were randomly sampled, with 30 quadrats plots (100 m^2^, divided into four subplots), per island. Surveys took place in spring and summer of 2017 (São Miguel and Terceira Islands) and 2018 (São Miguel and Pico Islands), for a period of 8 months (4 months per year), corresponding to a total of 240 days. Study areas were delimited using a geographic information system (GIS; QGis 3.28) to map and select forest stands, based on the data provided by the Azorean Forest Inventory (DRRF 2007) (see Borges Silva L et al. (2022b) , Figure 2).

### Funding

This work was funded by: i) Project FOREST-ECO2-Towards an Ecological and economic valorization of the Azorean Forest ACORES-01-0145-FEDER-000014-Azores 2020 PO, 2016–2019; ii) FEDER funds through the Operational Programme for Competitiveness Factors-COMPETE; iii) by PO Azores Project “Portal da Biodiversidade dos Açores” - M1.1.A/INFRAEST CIENT/001/2022; and iv) by National Funds through FCT-Foundation for Science and Technology under the UID/BIA/50027/2019 and POCI-01-0145-FEDER-006821.

## Sampling methods

### Study extent

Three types of vegetation were included (Borges Silva L et al. 2022):

- Natural forest, corresponding to submontane and montane cloud forests (Elias et al. 2016). Its distribution in the Azores evolved in unique conditions, due to a pronounced isolation, relatively homogeneous climatic conditions and a limited number of native woody species, but high plant biodiversity and a high number of endemic species, which are dominated by *Ilexazorica* Gand., *Juniperusbrevifolia* (Hochst. ex Seub.) Antoine, *Laurusazorica* (Seub.) Franco, *Morellafaya* (Aiton) Wilbur and *Picconiaazorica* (Tutin) Knobl (Dias et al. 2007, Elias and Dias 2008, Silva et al. 2010, Pavão et al. 2022, Pavão et al. 2023a, Pavão et al. 2023b).
- Exotic woodland, located at lower to mid-elevations and dominated by *P.undulatum* covering more than 30% of the forest area, which expanded from sea level up to 600 m (Borges Silva et al. 2017, Dutra Silva L et al. 2017a, Dutra Silva et al. 2017b, Borges Silva et al. 2018, Dutra Silva et al. 2019).
- Production forest, dominated by *C.japonica*, occupying 60% of the area dedicated to this type of forest, the most economically important forestry species in the Azores and with an important impact on the landscape (DRRF 2007, Lourenço et al. 2011, DRRF 2014).

### Sampling description

A total of 90 forest stands were randomly sampled, 30 in each of the three selected islands São Miguel, Terceira and Pico (10 NF, 10 EW and 10 PF) (Table 1). At each forest, we delimited a 10 × 10 m (100 m^2^) plot and recorded the vascular plant taxa. To quantify the cover-abundance of each taxon, we used the Braun Blanquet scale modified by Van der Maarel E (1979).

**Analysis**

*Colonisation status*. We determined the indigenous and non-indigenous plants globally and specifically for the groups Magnoliopsida, Liliopsida, Pinophyta, Pteridophyta and Lycopodiophyta. The contribution of each family was evaluated by calculating the number of genera for indigenous and non-indigenous taxa within each family and the number of infrageneric taxa per genus.

*Biogeography of non-indigenous plants.* The distribution of non-indigenous taxa was classified by region, according to Pielou (1992). Taxa present in more than two biogeographic regions were considered as subcosmopolitan.

*Life forms.* The classification of life forms followed Franco (1971), Franco (1984), Franco and Afonso (1994) and Franco and Afonso (1998) and the frequency of each life form was then calculated. Life forms were based on the Raunkjaer (1936) main criterion (height of perennating buds): phanerophytes-perennating buds on aerial shoots (nanophanerophytes *<* 2 m in height, microphanerophytes 2-8 m, mesophanerophytes 8-30 m, megaphanerophytes *>* 30 m); chamaephytes - perennating buds very close to the ground; hemicryptophytes - perennating buds at ground level; cryptophytes - perennating buds below ground level (geophytes) and therophytes - annual species (Silva and Smith 2004).

*Useful species*. The number of taxa in the following categories was calculated: ornamental, forestry, cultivated (aromatic, animal fodder, hedge-plants), crops (human food) and ruderal. The percentages of taxa considered as plant invaders and as ecological threats by Portuguese legislation were also calculated DLR 15 from 2 April 2012 (DLR15 2012) and DL 92 from 10 July 2019 (DL92 2019).

### Quality control

Specimens representing most of the inventoried species, were collected in the field, following standard herbarium techniques and then deposited in the Herbarium Ruy Telles Palhinha, University of the Azores (AZB). All sampled individuals were sorted by trained taxonomists.

Taxonomic nomenclature obtained from:Seubert and Hochstetter (1843), Seubert (1844), Dröuet (1866), Trelease (1897), Cedercreutz (1941), Tutin et al. (1964), Palhinha (1966), Tutin et al. (1968), Franco (1971), Tutin et al. (1972), Sjögren (1973), Tutin et al. (1976), Tutin et al. (1980), Fernandes and Fernandes (1980), Fernandes and Fernandes (1983), Franco (1984), Fernandes and Fernandes (1987), Valdés et al. (1987), Franco and Afonso (1994), Press and Short (1994), Franco and Afonso (1998), Schaefer (2002), Schaefer (2003), Schaefer (2005), Schaefer (2005a), Silva et al. (2010), Vieira et al. (2020)

In terms of species colonisation status, we followed Silva et al. (2010) categories: Azorean endemic species, i.e. species (or subspecies) occurring only in the Azores, as a result of either speciation events (neo-endemics) or extinct of the mainland populations (paleo-endemics); Macaronesian endemic species, i.e. species only known from the Macaronesian archipelagos (the Azores, Madeira, Canaries and Cape Verde); Native species, i.e. species which arrived by long-distance dispersal to the Azores and which also occur naturally elsewhere. Regarding Introduced species, that occur in the archipelago as a result of human activities, we distinguished two groups, Naturalised, with self-supporting populations and Casual, occasionally escaped from cultivation.

The biogeographic and historic criteria used to classify taxa as non-indigenous were adapted from Silva et al. (2000): (i) classified as such by several authors from the 19^th^ century; (ii) first record in the last 100 years; (iii) distribution restricted to a reduced number of islands; (iv) record of a recent (last 100 years) extension of the distribution in the Azores; (v) absence in other Macaronesian islands; (vi) disjunct distribution; (vii) anthropochoric taxa - only casual in the native vegetation. These criteria were applied after exclusion of endemic taxa. Database queries allowed the determination of the taxa number in each category, globally and specifically for the Magnoliopsida, Liliopsida, Pinophyta, Pteridophyta and Lycopodiophyta groups (Silva and Smith 2004).

## Geographic coverage

### Description

São Miguel, Terceira and Pico Islands, in the Azores Archipelago (Portugal).

**Coordinates**: São Miguel: 37°55'45.6''N and 37°42'22.8''N Latitude; 25°53'28.2''W and 25°0'27.6''W Longitude Terceira: 38°38'16.8''N and 38°48'50.4''N Latitude; 27°23'38.4''W and 27°0'54''W Longitude Pico: 38°34'53''N and 38°21'48''N Latitude; 28°33'40''W and 28°0'14.9''W Longitude.

## Taxonomic coverage

### Description

**Figure 3.**
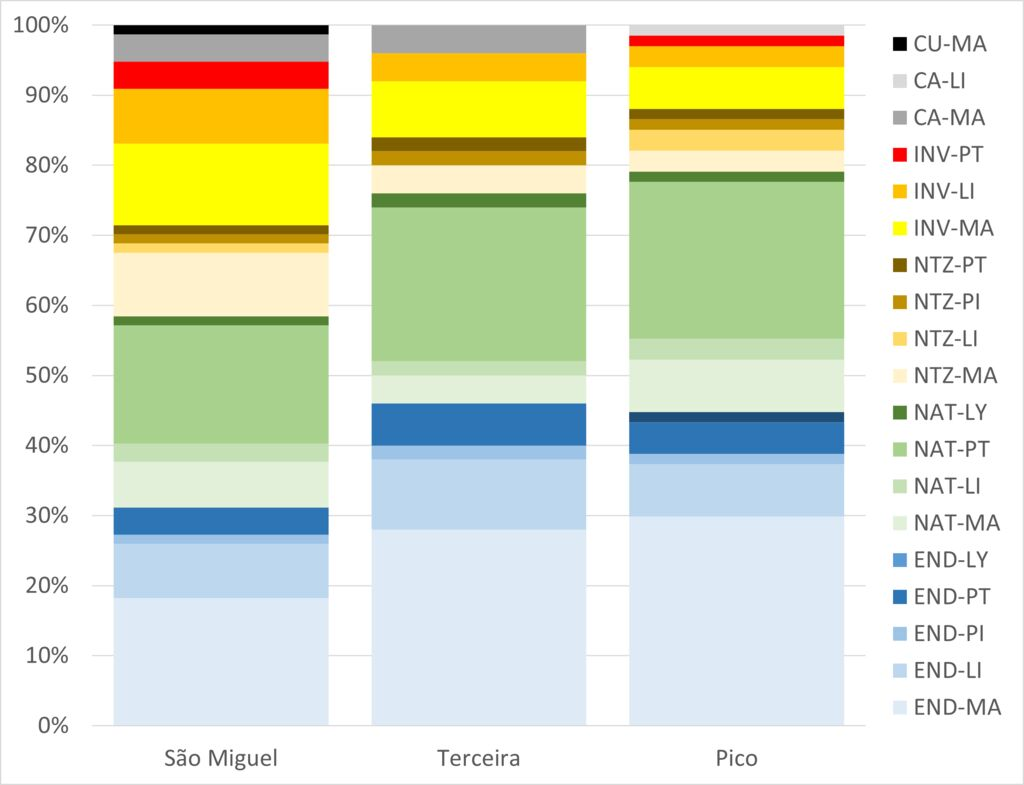
Categorisation of 105 Azorean vascular plant taxa, observed at 90 forests in the Azores, per Island (São Miguel, Terceira and Pico) and according to the five major groups of vascular plants (MA-Magnoliopsida; LI-Liliopsida; PI-Pinophyta; PT- Pteridophyta; and LY-Lycopodiophyta). Number of taxa in each category: END-Endemic (taxa only occurring in the Azores); NAT-Native (colonised Azores without human intervention, also occurring in other regions); INV-Invasive; NTZ-Naturalised (with self-supporting populations); CA-casual (occasionally escaped from cultivation) and CU-Cultivated (Silva et al. 2010).

For the three forest types and for the three Islands, the dataset includes 105 vascular plant taxa, represented by 101 species and four subspecies, mostly including indigenous plants (35% endemic and 27% native) and 38% of non-indigenous plants (Fig. 2 and Fig. 3). Magnoliopsida were the highest proportion and Pinophyta the lowest (Fig. 2 and Fig. 3). The 105 taxa were distributed by 60 families belonging to 91 genera (Fig. 2). In general, each family and genus contributed only with a small number of indigenous (1-6) or non-indigenous taxa (1-3) (Fig. 2).

Regarding Magnoliopsida, NF showed the highest numbers of endemic and native taxa, while EW and PF showed the highest values of invasive taxa, EW also showing a high number of naturalised and casual species (Fig. 2). For Liliopsida, again NF showed the highest values of endemism, despite also including naturalised and invasive taxa (Fig. 2). For Pteridophyta, all three forests showed similar values of endemic, native and invasive taxa (Fig. 2).

In the Pteridophyta, for the three forest types, 10 families included one or two genera, one family included three genera and only two families participated with more than two species, namely the Dryopteridaceae and Hymenophyllaceae (Table 2). We found two Lycophytes, one a Macaronesian endemism, *Huperziasuberecta* (Lowe) Tardieu. The Pinophyta only included two Cupressaceae, with one endemic taxon, Juniperusbrevifolia(Hochst. ex Seub.)Antoinesubsp.brevifolia (Table 2). For the three forest types, the Liliopsida included 11 families and 27 genera, most contributing with only one taxon. The Cyperaceae and Poaceae included the highest numbers of indigenous taxa (Table 2). For the Magnoliopsida, we found 35 families and 83 genera. In general, the families including more indigenous taxa were the Asteraceae, Ericaceae, Lauraceae and Rosaceae (Table 2).

The frequency of indigenous vascular plant taxa showed the highest values for NF (55% endemic and 38% native) and the lowest for EW (23% endemic and 25% native) and PF (17% endemic and 18% native) (Table 3). Pico Island displayed the highest number of indigenous taxa in the NF and the lowest number in PF (Table 3).

Regarding the conservation status of indigenous vascular plant taxa, we found 23 considered as Least Concern (LC) and two as Endangered (EN) in NF and in the three forest types, two as Vulnerable (VU) and two as Near Threatened (NT) (Table 3). We also found particularly rare species such as *Lactucawatsoniana* Trel. In total, 36 indigenous species (55%) had not been evaluated by IUCN criteria (Table 3).

Although, based on DLR no. 15/2012/A (DLR15 2012), 35% of the indigenous species in the current study are covered by measures for conservation and protection (Table 3), of these 60% are proprietary for conversion (P) and all taxa present in NF, 13% EW and 9% PF (Table 3).

## Traits coverage

**Life forms**

The majority of indigenous and non-indigenous Pteridophyta were hemicryptophytes, 60% and 75%, respectively, while Pinophyta only included one megaphanerophyte and one microphanerophyte.

**Figure 4.**
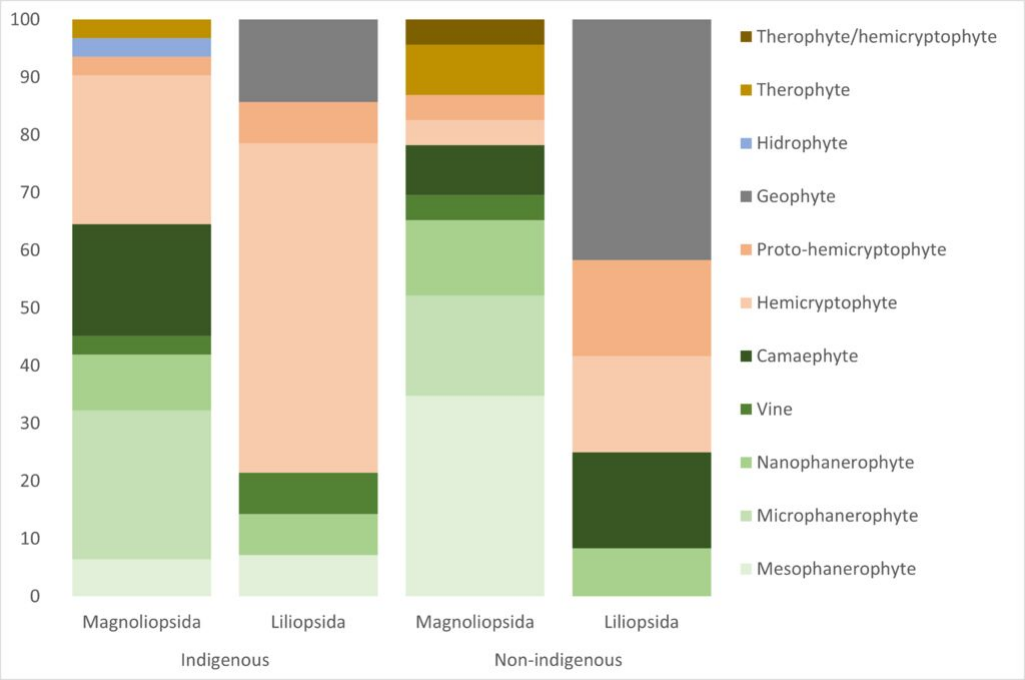
Frequency (%) of life forms for indigenous and non-indigenous Magnoliopsida and Liliopsida, for the three forest types and the three Islands (90 forests in the Azores).

Non-indigenous and indigenous Magnoliopsida included a larger proportion of phanerophytes than Liliopsida (Fig. 4), while geophytes and hemicryptophytes were of some importance in the Liliopsida (Fig. 4).

**Biogeography of non-indigenous plants**

**Figure 5.**
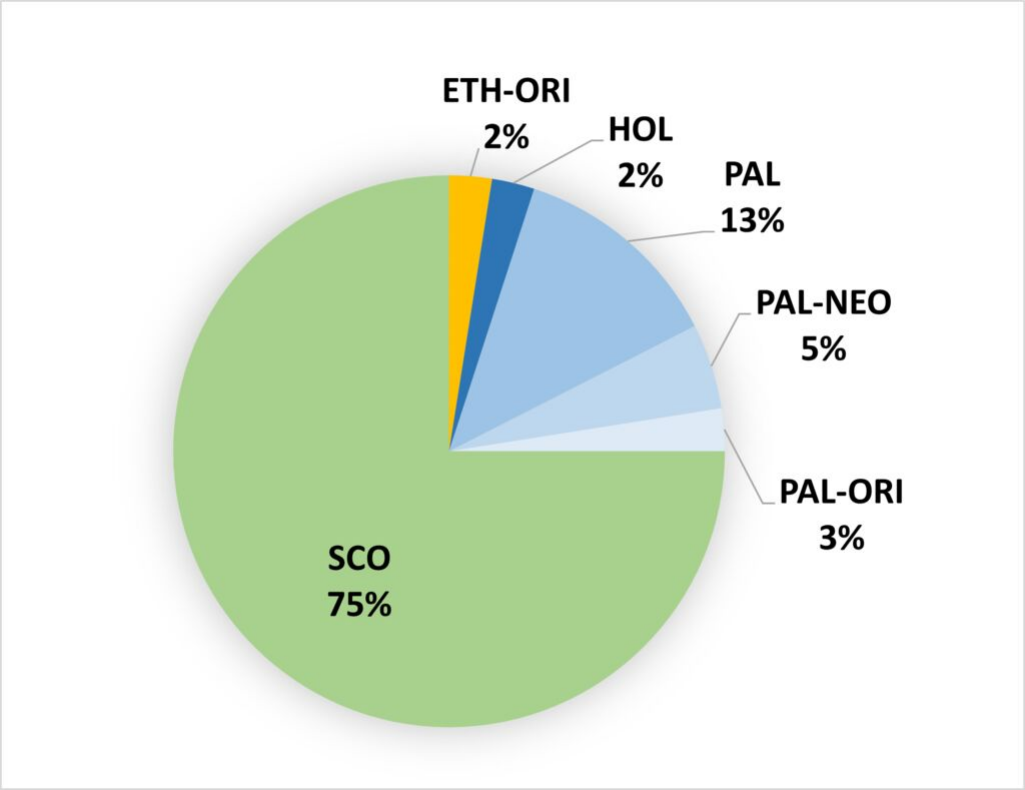
Biogeography of 40 non-indigenous vascular plant taxa sampled at 90 forests in São Miguel, Terceira and Pico Islands, Azores. SCO - Subcosmopolitan, NEO - Neotropical, PAL - Palaearctic, ORI - Oriental, ETH - Ethiopian, HOL – Holarctic. (Example: PALNEO - Palaearctic and Neotropical).

Most non-indigenous taxa had a wide geographic distribution. About 75% were Subcosmopolitan and a considerable percentage had a Palaearctic distribution (Fig. 5).

### Useful species

**Figure 6.**
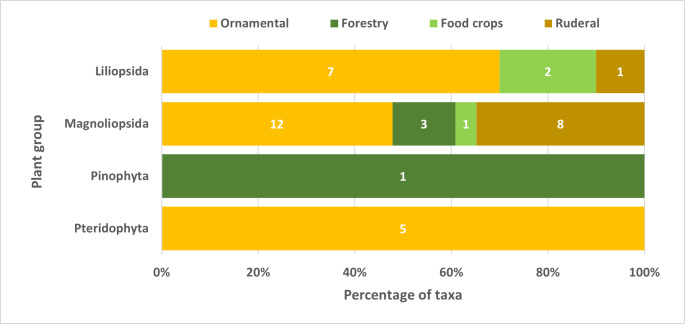
Percentage of non-indigenous vascular plants (naturalised or casual) with human utilisation (ornamental, forest species and food crops) or ruderal, for the three forest types in São Miguel, Terceira and Pico Islands, Azores. The data labels correspond to the number of taxa.

Almost all non-indigenous Pteridophyta were ornamental plants, while the Pinophyta were forest species (Fig. 6). The Magnoliopsida included a large proportion of ornamental and ruderal plants. The Liliopsida included a large percentage of ornamental and a small proportion of ruderal taxa (Fig. 6).

## Temporal coverage

### Notes

2017-4-09 - 2018-7-27

## Usage licence

### Usage licence

Creative Commons Public Domain Waiver (CC-Zero)

## Data resources

### Data package title

Vascular plant taxa occurrences in exotic woodland and in natural and production forests on the Islands of São Miguel, Terceira and Pico (Azores).

### Resource link

https://www.gbif.org/dataset/158e0f0d-26e3-4883-bf5e-6040c1bb1ff4

### Alternative identifiers

http://ipt.gbif.pt/ipt/resource?r=plants-azo-foresteco2&v=1.2

### Number of data sets

2

### Data set 1.

#### Data set name

Event Table

#### Data format

Darwin Core Archive format

#### Character set

UTF-8

#### Download URL

http://ipt.gbif.pt/ipt/resource?r=plants-azo-foresteco2&v=1.2

#### Data format version

Version 1.2

#### Description

The dataset was published in Global Biodiversity Information Facility platform, GBIF (Borges Silva et al. 2023).The following data table includes all the records for which a taxonomic identification of the species was possible. The dataset submitted to GBIF is structured as a sample event dataset that has been published as a Darwin Core Archive (DwCA), which is a standardised format for sharing biodiversity data as a set of one or more data tables. The core data file contains 90 records (eventID). This IPT (integrated publishing toolkit) archives the data and thus serves as the data repository. The data and resource metadata are availabe for download from Borges Silva et al. (2023).

**Data set 1.** 

Column label	Column description
eventID	Identifier of the events, unique for the dataset. https://dwc.tdwg.org/terms/#dwc:eventID
datasetName	The name identifying the data set from which the record was derived. https://dwc.tdwg.org/terms/#dwc:datasetName
habitat	The habitat for an Event. https://dwc.tdwg.org/terms/#dwc:habitat
samplingProtocol	The sampling protocol used to capture the species. https://dwc.tdwg.org/terms/#dwc:samplingProtocol
sampleSizeValue	The numeric amount of time spent in each sampling. https://dwc.tdwg.org/terms/#dwc:sampleSizeValue
sampleSizeUnit	The unit of the sample size value. https://dwc.tdwg.org/terms/#dwc:sampleSizeUnit
samplingEffort	The amount of time of each sampling. https://dwc.tdwg.org/terms/#dwc:samplingEffort
eventDate	Date of the sampling. https://dwc.tdwg.org/terms/#dwc:eventDate
locationID	Identifier of the location. https://dwc.tdwg.org/terms/#dwc:locationID
islandGroup	Name of the archipelago. https://dwc.tdwg.org/terms/#dwc:islandGroup
island	Name of the island. https://dwc.tdwg.org/terms/#dwc:island
country	Country of the sampling site. https://dwc.tdwg.org/terms/#dwc:county
countryCode	The standard code for the country of the sampling site. https://dwc.tdwg.org/terms/#dwc:countryCode
stateProvince	Name of the region of the sampling site. https://dwc.tdwg.org/terms/#dwc:stateProvince
municipality	Municipality of the sampling site. https://dwc.tdwg.org/terms/#dwc:municipality
locality	Name of the locality. https://dwc.tdwg.org/terms/#dwc:locality
locationRemarks	Comments or notes about the Location. https://dwc.tdwg.org/terms/#dwc:locationRemarks
minimumElevationInMetres	The lower limit of the range of elevation (altitude, usually above sea level), in metres. https://dwc.tdwg.org/terms/#dwc:minimumElevationInMeters
maximumElevationInMetres	The upper limit of the range of elevation (altitude, usually above sea level), in metres. https://dwc.tdwg.org/terms/#dwc:maximumElevationInMeters
verbatimCoordinates	Original coordinates recorded. https://dwc.tdwg.org/terms/#dwc:verbatimCoordinates
decimalLatitude	Approximate centre point decimal latitude of the field site in GPS coordinates. https://dwc.tdwg.org/terms/#dwc:decimalLatitude
decimalLongitude	Approximate centre point decimal longitude of the field site in GPS coordinates. https://dwc.tdwg.org/terms/#dwc:decimalLongitude
geodeticDatum	The ellipsoid, geodetic datum or spatial reference system (SRS) upon which the geographic coordinates given in decimalLatitude and decimalLongitude are based. https://dwc.tdwg.org/terms/#dwc:geodeticDatum
coordinateUncertaintyInMetres	Uncertainty of the coordinates of the centre of the sampling plot in metres. https://dwc.tdwg.org/terms/#dwc:coordinateUncertaintyInMeters
georeferenceSources	Method used to obtain coordinates. https://dwc.tdwg.org/terms/#dwc:georeferenceSources

### Data set 2.

#### Data set name

Occurrence Table

#### Data format

Darwin Core

#### Character set

UTF-8

#### Download URL

http://ipt.gbif.pt/ipt/resource?r=plants-azo-foresteco2&v=1.2

#### Data format version

Version 1.2

#### Description

The dataset was published in Global Biodiversity Information Facility platform, GBIF (Borges Silva et al. 2023). The following data table includes all the records for which a taxonomic identification of the species was possible. The dataset submitted to GBIF is structured as a occurrence table that has been published as a Darwin Core Archive (DwCA), which is a standardised format for sharing biodiversity data as a set of one or more data tables. The core data file contains 1150 records (occurrenceID). This IPT (integrated publishing toolkit) archives the data and thus serves as the data repository. The data and resource metadata are available for download from Borges Silva et al. (2023).

**Data set 2.** 

Column label	Column description
licence	Reference to the licence under which the record is published. https://dwc.tdwg.org/terms/#dcterms:license
institutionID	The identity of the institution publishing the data. https://dwc.tdwg.org/terms/#dwc:institutionID
institutionCode	The code of the institution publishing the data. https://dwc.tdwg.org/terms/#dwc:institutionCode
basisOfRecord	The nature of the data record. https://dwc.tdwg.org/terms/#dwc:basisOfRecord
occurrenceID	Identifier of the record, coded as a global unique identifier. https://dwc.tdwg.org/terms/#dwc:occurrenceID
recordedBy	A list of names of the people who performed the sampling of the specimens. https://dwc.tdwg.org/terms/#dwc:recordedBy
organismQuantity	A number or enumeration value for the quantity of organisms. https://dwc.tdwg.org/terms/#dwc:organismQuantity
organismQuantityType	The type of quantification system used for the quantity of organisms. https://dwc.tdwg.org/terms/#dwc:organismQuantityType
establishmentMeans	The process of establishment of the species in the location, using a controlled vocabulary: 'native', 'introduced'. https://dwc.tdwg.org/terms/#dwc:establishmentMeans
eventID	Identifier of the events, unique for the dataset. https://dwc.tdwg.org/terms/#dwc:eventID
identifiedBy	A list of names of people who assigned the Taxon to the subject. https://dwc.tdwg.org/terms/#dwc:identifiedBy
dateIdentified	Date on which the record was identified. https://dwc.tdwg.org/terms/#dwc:dateIdentified
scientificName	Complete scientific name including author. https://dwc.tdwg.org/terms/#dwc:scientificName
kingdom	Kingdom name. https://dwc.tdwg.org/terms/#dwc:kingdom
phylum	Phylum name. https://dwc.tdwg.org/terms/#dwc:phylum
class	Class name. https://dwc.tdwg.org/terms/#dwc:class
order	Order name. https://dwc.tdwg.org/terms/#dwc:order
family	Family name. https://dwc.tdwg.org/terms/#dwc:family
genus	Genus name. https://dwc.tdwg.org/terms/#dwc:genus
specificEpithet	Specific epithet. https://dwc.tdwg.org/terms/#dwc:specificEpithet
infraspecificEpithet	Infraspecific epithet, when available. https://dwc.tdwg.org/terms/#dwc:infraspecificEpithet
taxonRank	Lowest taxonomic rank of the record. https://dwc.tdwg.org/terms/#dwc:taxonRank
scientificNameAuthorship	Name of the author of the lowest taxon rank included in the record.https://dwc.tdwg.org/terms/#dwc:scientificNameAuthorship
taxonRemarks	Comments or notes about the taxon or name. https://dwc.tdwg.org/terms/#dwc:taxonRemarks
dynamicProperties	A list of additional measurements, facts, characteristics or assertions about the record. Meant to provide a mechanism for structured content. https://dwc.tdwg.org/terms/#dwc:dynamicProperties

## Additional information

**Conclusions and prospects**

For the three forest types and for all Islands, the dataset included 105 vascular plant taxa, 62% indigenous and 38% non-indigenous, distributed by 60 families belonging to 91 genera, each family and genus contributing only with a small number of taxa.

Regarding Magnoliopsida, NF showed the highest number of endemic and native taxa, while EW and PF showed the highest values of invasive taxa, with EW also showing a high number of naturalised and casual taxa. For Liliopsida, again NF showed the highest values of endemism, despite also including naturalised and invasive taxa. For Pteridophyta, all three forest types showed similar values of endemic, native and invasive taxa.

The frequency of indigenous vascular plant taxa was highest for NF (55% endemic and 38% native) and lowest for EW (23% endemic and 25% native) and PF (17% endemic and 18% native). Pico Island displayed the highest number of indigenous species in NF and the lowest number in PF. The results of this study agree with data from previous investigations (Borges Silva L et al. 2022b). As expected, natural forests correspond to indigenous plant diversity hotspots and exotic woodland works as a source of invasive taxa.

According to our results, 35% of the indigenous plant taxa in the current study are covered by conservation regulations.

The list of the vascular plants found in our study devoted to natural and production forests and to exotic woodland in three Azores Islands, clarifies the type of flora to be expected in the forested areas in the Azores, emphasising the relevant role of the former as hotspots of native biodiversity, which agrees with previous studies for the Azores (Silva et al. 2010, Marcelino et al. 2013, Mendonça 2013, Marcelino et al. 2014, Elias et al. 2016, Borges 2018, Borges Silva et al. 2018, Borges Silva L et al. 2022b).

As stated by Borges Silva L et al. (2022b), natural forests mainly corresponded to montane forests which occur in the thermotemperate-hyperhumid and ultrahyperhumid belts, from 600 to 1000 m a.s.l., in areas with high rainfall (3000 to 5000 mm year^−1^) and occult precipitation (cloud forests) (Gabriel and Bates 2005, Elias et al. 2016). These forests have small stature and are frequently subjected to natural disturbances (Elias et al. 2011). This favours plant diversity by allowing the existence of both light-demanding and shade tolerant species. They are characterised by a high percentage of endemic species, trees covered by epiphytes and a complex vertical structure with several layers (Elias et al. 2016).

Meanwhile, the role of exotic woodland and, to a lesser extent, of production forest, as reservoirs of invasive species is also confirmed (Borges Silva L et al. 2022b). The low plant diversity noted in EW and PF could be explained by the dominance of a single species (*Pittospotumundulatum* and *Cryptomeriajaponica*, respectively), contributing with nearly 90% of the total number of trees per plot and dominating the canopy where only ferns and a few invasive species that tolerate low levels of light intensities below 1% full sunlight at ground level (e.g. *Hedychiumgardnerianum*) are found (Gleadow and Ashton 1981, Gleadow et al. 1983, Cordeiro and Silva 2003, Borges Silva L et al. 2022b).

Regarding anthropogenic action, NF have less human influence and are hard to access (Ramos 1996). Disturbances are limited to minor harvesting of non-timber forest products (Borges Silva L et al. 2022b). Nevertheless, active and persistent conservation measures are needed to ensure the preservation of the natural forests in the Azores. In the case of PF and the spread of invasive species in EW, an intensive management regime resulted in a decrease in plant diversity levels (Silva et al. 2008, Castro et al. 2010, Kueffer et al. 2010, Gil et al. 2013, DRRF 2017). While plantations are known for high timber productivity, their potential to harbour plant diversity is low (Borges et al. 2019). In the Azores, the new production forests already include a buffer zone with native elements (DRRF 2017). However, management plans should be developed for exotic woodland, following the guidelines already established for the renovation of *Cryptomeriajaponica* production forests in the Azores.

Finally, we consider that our dataset and the derived conclusions will be useful for future conservation and research activities, as well as for forest managers, in the development of more comprehensive action plans, particularly on islands.

## Figures and Tables

**Table 1.** List of the 90 sampled forests in São Miguel (SMG, n = 30), Terceira (TER, n = 30) and Pico (PIC, n = 30) Islands. Information about Forest Type (Natural - Natural Forest, Exotic-*Pitt* - Exotic Woodland dominated by *Pittosporumundulatum*, Production-*Cryp* - Production Forest dominated by *Cryptomeriajaponica*), Location ID (Code), Locality, decimal geographical coordinates (datum WGS84) and elevation in metres is provided.

**Island**	**Forest type**	**Location ID**	**Locality**	**Latitude**	**Longitude**	**Elevation (m)**
SMG	Natural	SMLO-NF01	Lombadas	37.78824	-25.46862	654
SMG	Natural	SMTR-NF02	Tronqueira	37.79914	-25.18354	646
SMG	Natural	SMVO-NF03	Lomba do Carro	37.78122	-25.27603	568
SMG	Natural	SMER-NF04	Sete Cidades (Estrada-Sul Vista do Rei)	37.83654	-25.78826	640
SMG	Natural	SMLF-NF05	Lagoa do Fogo	37.76259	-25.46632	653
SMG	Natural	SMVA-NF06	Pico da Vela	37.76174	-25.46140	855
SMG	Natural	SMMT-NF07	Tronqueira	37.79804	-25.18430	677
SMG	Natural	SMME-NF08	Monte Escuro	37.77866	-25.43709	753
SMG	Natural	SMTR-NF09	Tronqueira	37.79701	-25.18442	754
SMG	Natural	SMLM-NF10	Lomba do Botão	37.77383	-25.27546	455
SMG	Exotic-*Pitt*.	SMCM-EW01	Ferraria (Pico das Camarinhas)	37.85843	-25.84877	205
SMG	Exotic-*Pitt*.	SMCB-EW02	Lagoa (Chã do Rego d`Água)	37.77367	-25.56959	240
SMG	Exotic-*Pitt*.	SMMF-EW03	Mosteiros (Pico de Mafra)	37.89435	-25.80720	200
SMG	Exotic-*Pitt*.	SMLS-EW04	Lagoa Santiago	37.85266	-25.77467	502
SMG	Exotic-*Pitt*.	SMPP-EW05	Pinhal da Paz	37.79015	-25.63265	394
SMG	Exotic-*Pitt*.	SMFA-EW06	Fenais da Ajuda (Lomba de Cima)	37.82921	-25.31140	405
SMG	Exotic-*Pitt*.	SMFT-EW07	Faial da Terra	37.75355	-25.21646	508
SMG	Exotic-*Pitt*.	SMPB-EW08	Pico Bartolomeu	37.80110	-25.15830	607
SMG	Exotic-*Pitt*.	SMFN-EW09	Furnas Norte (Caminho Norte/Sul)	37.78404	-25.29760	315
SMG	Exotic-*Pitt*.	SMNG-EW10	Lagoa do Congro	37.75813	-25.40702	607
SMG	Production-*Cryp*.	SMAL-PF01	Caldeira do Alferes	37.87024	-25.80575	483
SMG	Production-*Cryp*.	SMEM-PF02	Lagoa das Empadadas	37.82668	-25.74983	845
SMG	Production-*Cryp*.	SMCT-PF03	Castelo Branco	37.74732	-25.35377	638
SMG	Production-*Cryp*.	SMFN-PF04	Furnas Norte (Caminho Norte/Sul)	37.77344	-25.36415	654
SMG	Production-*Cryp*.	SMAG-PF05	Água de Pau	37.73415	-25.49549	432
SMG	Production-*Cryp*.	SMPG-PF06	Planalto dos Graminhais	37.80362	-25.26304	850
SMG	Production-*Cryp*.	SMTR-PF07	Tronqueira	37.79797	-25.17647	683
SMG	Production-*Cryp*.	SMSB-PF08	Lagoa São Brás	37.79404	-25.41236	716
SMG	Production-*Cryp*.	SMFT-PF09	Faial da Terra	37.77265	-25.18795	470
SMG	Production-*Cryp*.	SMGI-PF10	Ginetes	37.87205	-25.82743	289
TER	Natural	TETB-NF01	Reserva Natural da Terra Brava	38.73293	-27.20936	692
TER	Natural	TEBL-NF02	Reserva Natural da Terra Brava	38.73942	-27.21194	710
TER	Natural	TETR-NF03	Reserva Natural da Terra Brava	38.73086	-27.19350	679
TER	Natural	TETA-NF04	Reserva Natural da Terra Brava	38.73008	-27.19260	692
TER	Natural	TEIN-NF05	Caldeira de Santa Bárbara	38.73461	-27.30828	889
TER	Natural	TEIS-NF06	Caldeira de Santa Bárbara	38.73390	-27.30933	904
TER	Natural	TEMO-NF07	Morro Assombrado	38.75619	-27.22497	591
TER	Natural	TEMA-NF08	Morro Assombrado	38.75763	-27.22706	550
TER	Natural	TELM-NF09	Lomba	38.73911	-27.29289	725
TER	Natural	TELO-NF10	Lomba	38.73844	-27.29036	700
TER	Exotic-*Pitt*.	TEMD-EW01	Monte Brasil (Ponta de São Diogo)	38.64105	-27.22841	212
TER	Exotic-*Pitt*.	TESE-EW02	Serreta (Pico do Carneiro)	38.76431	-27.35234	492
TER	Exotic-*Pitt*.	TESC-EW03	Biscoitos	38.79195	-27.24367	139
TER	Exotic-*Pitt*.	TEPT-EW04	Pico do Teles	38.73238	-27.36180	443
TER	Exotic-*Pitt*.	TEFE-EW05	Feteira	38.66165	-27.15121	279
TER	Exotic-*Pitt*.	TEMG-EW06	Caparica (Caminho dos Caneleiros)	38.77158	-27.26219	386
TER	Exotic-*Pitt*.	TELJ-EW07	Vila das Lajes	38.75774	-27.10938	129
TER	Exotic-*Pitt*.	TECU-EW08	Serra do Cume	38.72935	-27.09827	264
TER	Exotic-*Pitt*.	TELC-EW09	São Brás (Ladeira do Cardoso)	38.74693	-27.13439	265
TER	Exotic-*Pitt*.	TEAG-EW10	Agualva	38.79077	-27.19684	197
TER	Production-*Cryp*.	TETE-PF01	Terra Chã	38.69952	-27.23730	517
TER	Production-*Cryp*.	TESS-PF02	Serra de Santa Bárbara	38.71403	-27.32952	600
TER	Production-*Cryp*.	TEGN-PF03	Gruta de Natal	38.73102	-27.28399	661
TER	Production-*Cryp*.	TEGR-PF04	Gruta de Natal	38.73999	-27.26335	593
TER	Production-*Cryp*.	TEBI-PF05	Biscoitos	38.76861	-27.25169	443
TER	Production-*Cryp*.	TECB-PF06	São Bento (Caminho do Cabrito)	38.70378	-27.17663	468
TER	Production-*Cryp*.	TEEC-PF07	Algar do Carvão (Caminho)	38.72588	-27.24099	579
TER	Production-*Cryp*.	TEMH-PF08	Malha Grande	38.75945	-27.26404	498
TER	Production-*Cryp*.	TERF-PF09	Reserva Florestal Parcial (Serreta e Serra de Santa Bárbara)	38.76519	-27.32107	555
TER	Production-*Cryp*.	TEMS-PF10	Mato da Serreta	38.74679	-27.33689	800
PIC	Natural	PISG-NF01	Saída das Lages	38.43333	-28.30689	419
PIC	Natural	PIPR-NF02	Mistério da Prainha	38.48544	-28.27356	516
PIC	Natural	PIBU-NF03	Trilho dos Burros	38.47972	-28.27231	621
PIC	Natural	PIAC-NF04	Planalto da Achada	38.46914	-28.31014	682
PIC	Natural	PICD-NF05	Caiado	38.45589	-28.25708	808
PIC	Natural	PICA-NF06	Caveiro	38.43753	-28.20108	905
PIC	Natural	PICT-NF07	Caveiro	38.43606	-28.20761	940
PIC	Natural	PIAF-NF08	Caminho do Arrife	38.45067	-28.30986	580
PIC	Natural	PICS-NF09	Cabeçinhos	38.44350	-28.31883	530
PIC	Natural	PICX-NF10	Cabeço do Teixo	38.48775	-28.34708	850
PIC	Exotic-*Pitt*.	PIPH-EW01	Prainha	38.46781	-28.21922	296
PIC	Exotic-*Pitt*.	PISR-EW02	São Roque	38.51144	-28.33072	282
PIC	Exotic-*Pitt*.	PISL-EW03	Santa Luzia	38.52550	-28.39169	344
PIC	Exotic-*Pitt*.	PIAM-EW04	Santo Amaro	38.45053	-28.18022	229
PIC	Exotic-*Pitt*.	PIPE-EW05	Piedade	38.43203	-28.07394	200
PIC	Exotic-*Pitt*.	PIRI-EW06	Ribeiras	38.41289	-28.14211	358
PIC	Exotic-*Pitt*.	PILG-EW07	Lajes do Pico	38.42542	-28.27253	288
PIC	Exotic-*Pitt*.	PICB-EW08	Candelária	38.47233	-28.50047	124
PIC	Exotic-*Pitt*.	PIBD-EW09	Bandeiras	38.52964	-28.46250	202
PIC	Exotic-*Pitt*.	PIJO-EW10	São João	38.42733	-28.33086	320
PIC	Production-*Cryp*.	PIFR-PF01	Farrobo	38.51358	-28.43758	534
PIC	Production-*Cryp*.	PIBU-PF02	Trilho dos Burros	38.47986	-28.27272	627
PIC	Production-*Cryp*.	PIAR-PF003	São Miguel Arcanjo	38.49628	-28.29019	377
PIC	Production-*Cryp*.	PIAF-PF004	Caminho do Arrife	38.45461	-28.30689	604
PIC	Production-*Cryp*.	PIJO-PF005	São João	38.44089	-28.32081	514
PIC	Production-*Cryp*.	PICE-PF006	São Caetano	38.43192	-28.36583	434
PIC	Production-*Cryp*.	PIIR-PF007	Ribeirinhas	38.43044	-28.09308	362
PIC	Production-*Cryp*.	PIAM-PF008	Santo Amaro	38.44797	-28.15064	292
PIC	Production-*Cryp*.	PIPH-PF009	Prainha	38.46158	-28.21608	374
PIC	Production-*Cryp*.	PICI-PF010	Caminho do Caveiro (Lagoa do Caiado)	38.45689	-28.25922	804

**Table 2.** Number of genera and of infrageneric taxa per family, for indigenous and non-indigenous vascular plants, in three forest types (Natural Forest, Exotic Woodland and Production Forest), on three Azores Islands (São Miguel, Terceira and Pico). Numbers within brackets represent number of genera shared between indigenous and non-indigenous taxa.

**Family**	**Indigenous**		**Non-indigenous**		**Family**	**Indigenous**		**Non-indigenous**	
	**Genera**	**Taxa**	**Genera**	**Taxa**		**Genera**	**Taxa**	**Genera**	**Taxa**
**Magnoliopsida**					**Liliopsida**				
Adoxaceae	1	1			Amaryllidaceae			1	1
Apiaceae	1	1			Araceae			2	2
Apocynaceae			1	1	Asparagaceae			1	1
Aquifoliaceae	1	1			Commelinaceae			1	1
Araliaceae	1	1			Cyperaceae	1	4		
Asteraceae	4	5	3	3	Iridaceae			1	1
Brassicaceae	1	1			Juncaceae	2(1)	2	1(1)	1
Caprifoliaceae			1	1	Orchidaceae	1	2		
Clethraceae			1	1	Poaceae	3(1)	3	2(1)	2
Ericaceae	3	3			Smilacaceae	1	1		
Euphorbiaceae	1	1			Zingiberaceae			1	1
Fabaceae			1	1	**Pinophyta**				
Geraniaceae			1	1	Cupressaceae	1	1	1	1
Hydrangeaceae			1	1	**Pteridophyta**				
Hypericaceae	1	1			Aspleniaceae	1	2		
Lamiaceae			1	1	Athyriaceae	2	2	1	1
Lauraceae	1(1)	1	3(1)	3	Blechnaceae	2	2	1	1
Myricaceae	1	1			Culcitataceae	1	1		
Myrsinaceae	1	1			Cyatheaceae			1	1
Myrtaceae			2	2	Dennstaedtiaceae	1	1		
Oleaceae	1	1			Dryopteridaceae	3	6	1	1
Onagraceae			1	1	Hymenophyllaceae	2	3		
Pittosporaceae			1	1	Osmundaceae	1	1		
Plantaginaceae	1	1			Polypodiaceae	1	1		
Platanaceae			1	1	Pteridaceae	1	1	1	1
Primulaceae	1	1			**Lycopodiophyta**				
Rhamnaceae	1	1			Lycopodiaceae	1	1		
Rosaceae	4 (1)	4	1(1)	1	Selaginellaceae	1	1		
Rubiaceae	1	1							
Santalaceae	1	1							
Scrophulariaceae	1	1							
Solanaceae			2	2					
Thymelaeaceae	1	1							
Ulmaceae			1	1					
Urticaceae			2	2					

**Table 3.** Conservation status and occurrence of indigenous vascular plant taxa per forest type (NF-Natural Forest, EW-Exotic Woodland and PF-Production Forest), in each Island (SMG-São Miguel; TER-Terceira and PIC-Pico). Based on 90 forests sampled in the Azores. Colonisation status (CS) follows Silva et al. (2010): NAT-Native and END-Endemic. Conservation status according to IUCN (threatened species: VU = Vulnerable, EN = Endangered; LC = Least Concern, NT = Near Threatened) and DLR no. 15/2012/A (Legal regime for nature conservation and protection of the Azores Autonomous Region biodiversity): H (Habitats Directive - Natura 2000 Network); B (Berne Convention); T100 (100 priority threatened species for management in the European biogeographical region of Macaronesia); CITES; R4 (protected by regional interest); P (priority for conservation) and *(priority European species).

Scientific Name	CS	Forest type			Island			Conservation				
		NF	EW	PF	SMG	TER	PIC	DLR15	H	B	UICN	Others
*Arceuthobiumazoricum* Wiens & Hawksw.	END	X				X	X	X	X			T100|P
*Aspleniumadiantum-nigrum* L.	NAT		X		X							
*Aspleniumscolopendrium* L.	NAT		X			X						
*Athyriumfilix-femina* (L.) Roth	NAT	X	X	X	X	X	X				LC	
*Bellisazorica* Hochst. ex Seub.	END	X					X	X		X		T100|P
*Callitrichestagnalis* Scop.	NAT	X					X				LC	
*Callunavulgaris* (L.) Hull	NAT	X			X						LC	
*Cardaminecaldeirarum* Guthnick ex Seub.	END	X			X							
*Carexdivulsa* Stokes	NAT	X	X		X	X	X					
*Carexhochstetteriana* J.Gay ex Seub.	END		X	X	X	X						
*Carexpendula* Huds.	NAT		X		X							
*Carexvulcani* Hochst. ex Seub.	END	X	X			X	X					
*Culcitamacrocarpa* C.Presl	NAT	X	X	X	X	X	X	X	X	X	NT	T100
*Daphnelaureola* L.	NAT	X					X					
*Deschampsiafoliosa* Hack.	END	X			X							
*Diplaziumcaudatum* (Cav.) Jermy	NAT	X	X	X	X	X	X				LC	
*Dryopterisaemula* (Aiton) Kuntze	NAT	X	X	X	X	X	X				LC	
*Dryopterisaffinis* (Lowe) Fraser-Jenk.	NAT	X					X					
*Dryopterisazorica* (Christ) Alston	END	X	X	X	X	X	X					
*Dryopteriscrispifolia* Rasbach, Reichst. & Vida	END	X	X	X	X	X	X				LC	
*Elaphoglossumhirtum* (Sw.) C.Chr.	NAT	X			X	X	X				EN	
*Ericaazorica* Hochst. ex Seub.	END	X	X		X	X	X	X	X	X		
EuphorbiastygianaH.C.Watsonsubsp.stygiana	END	X					X	X	X	X		T100|P
*Festucafrancoi* Fern.Prieto, C.Aguiar, E.Días & M.I.Gut	END	X			X							
*Fragariavesca* L.	NAT	X			X							
*Frangulaazorica* Grubov	END	X			X	X	X	X	X	X	LC	T100|P
*Hederaazorica* Carrière	END	X	X	X	X	X	X					
*Holcusrigidus* Hochst. ex Seub.	END	X			X	X						
*Huperziasuberecta* (Lowe) Tardieu	END	X					X	X	X		LC	R4
*Hymenophyllumtunbrigense* (L.) Sm.	NAT	X		X	X	X	X				LC	
*Hymenophyllumwilsonii* Hook.	NAT	X				X	X				LC	
*Hypericumfoliosum* Aiton	END	X			X	X	X				LC	
*Ilexazorica* Gand.	END	X	X	X	X	X	X	X			LC	T100
*Juncuseffusus* L.	NAT	X					X					
Juniperusbrevifolia(Hochst. ex Seub.)Antoinesubsp.brevifolia	END	X			X	X	X	X		X	VU	T100|P
*Lactucawatsoniana* Trel.	END	X					X	X	X	X	EN	T100|*
*Laurusazorica* (Seub.) Franco	END	X	X	X	X	X	X	X			LC	T100|P
*Leontodonfilii* (Hochst. ex Seub.) Paiva & Ormonde	END	X			X			X		X		T100|P
*Leontodonrigens* (Aiton) Paiva & Ormonde	END	X					X					
*Luzulapurpureosplendens* Seub.	END	X		X	X	X	X					
*Lysimachiaazorica* Hornem. ex Hook.	END	X	X	X	X	X	X					
*Morellafaya* (Aiton) Wilbur	NAT	X	X	X	X	X	X				LC	
*Myrsineretusa* Aiton	END	X	X	X	X	X	X					
*Osmundaregalis* L.	NAT	X			X		X				LC	
*Picconiaazorica* (Tutin) Knobl.	END	X	X			X	X	X	X	X	LC	T100|P
*Platantheramicrantha* (Hochst. ex Seub.) Schltr.	END	X					X	X				CITES|P
*Platantherapollostantha* R.M.Bateman & M.Moura	END	X					X					
Polypodiummacaronesicumsubsp.azoricum (Vasc.) Rumsey, Carine & Robba	END	X	X		X	X	X					
*Polystichumsetiferum* (Forssk.) T.Moore ex Woynar	NAT	X	X		X		X					
*Potentillaerecta* (L.) Raeusch.	NAT	X			X		X					
Prunuslusitanicasubsp.azorica (Mouill.) Franco	END	X			X			X	X	X		T100|P
*Pteridiumaquilinum* (L.) Kuhn	NAT	X	X	X	X	X	X				LC	
*Pterisincompleta* Cav.	NAT	X	X	X	X		X				NT	
*Rubiaagostinhoi* Dansereau & P.Silva	END	X	X	X	X	X	X					
*Rubushochstetterorum* Seub.	END	X					X	X			LC	P
*Saniculaazorica* Guthnick ex Seub.	END	X					X	X	X	X		T100|P
*Selaginellakraussiana* (Kunze) A.Braun	NAT	X	X	X	X	X	X				LC	
*Sibthorpiaeuropaea* L.	NAT	X	X	X	X	X	X					
*Smilaxazorica* H.Schaef. & P.Schönfelder	END	X	X		X	X	X	X		X		
*Struthiopterisspicant* (L.) Weis	NAT	X	X	X	X	X	X				LC	
*Tolpisazorica* (Nutt.) P.Silva	END	X				X	X	X				R4
*Vacciniumcylindraceum* Sm.	END	X	X	X	X	X	X	X			LC	T100|P
*Vandenboschiaspeciosa* (Willd.) G.Kunkel	NAT	X				X	X	x	X	X	LC	
*Viburnumtreleasei* Gand.	END	X			X	X	X	X			LC	T100|P
*Woodwardiaradicans* (L.) Sm.	NAT	X	X	X	X		X	X	X	X	VU	
